# Data Resource: population level family justice administrative data with opportunities for data linkage

**DOI:** 10.23889/ijpds.v5i1.1339

**Published:** 2020-06-09

**Authors:** RD Johnson, DV Ford, K Broadhurst, L Cusworth, KH Jones, A Akbari, S Bedston, B Alrouh, S Doebler, A Lee, J Smart, S Thompson, L Trinder, LJ Griffiths

**Affiliations:** 1 Population Data Science, Swansea University Medical School, Swansea, SA2 8PP, UK; 2 Centre for Child & Family Justice Research, Lancaster University, Lancaster, LA1 4YW, UK; 3 Law School, University of Exeter, Exeter, EX4 4RJ, UK

## Abstract

**Introduction:**

Although there has been considerable progress in the use of administrative data for applied health research, the family justice field lags behind. Better use of administrative data are essential to enhance understanding of how the family justice system is working, as well as the characteristics of, and outcomes for, children and families. The Family Justice Data Partnership (FJDP) supports this aim through analyses of core family justice and linked datasets in the SAIL Databank (Secure Anonymised Information Linkage).

Cafcass Cymru provide expert advice for children involved in family court proceedings in Wales, ensuring decisions are made in the best interests of the child. We provide an overview of Cafcass Cymru data. We also describe and illustrate linkage to administrative datasets within SAIL.

**Methods:**

Cafcass Cymru data was transferred to SAIL using a standardised approach to provide de-identified data with Anonymised Linking Fields (ALF) for successfully matched records. Three cohorts were created: all individuals involved in family court applications; all individuals with an ALF allowing subsequent health data linkage; and all individuals with a Residential Anonymised Linking Field (RALF) enabling area-level deprivation analysis.

**Results:**

Cafcass Cymru application data are available for child protection matters (public law, range 2011-2019, n=12,745), and child arrangement disputes (private law, range 2005-2019, n=52,023). An 80% data linkage match rate was achieved. 40% had hospital admissions within two years pre or post application; 54% had emergency department attendances and 61% had outpatient appointments. Individuals were more likely to reside in deprived areas regardless of law type.

**Conclusion:**

Cafcass Cymru data can be accessed through the SAIL Databank. The FJDP will continue to enhance research opportunities for all to better understand the family justice system, and outcomes for those involved, such as health and wellbeing for children and family members.

## Introduction

The family justice system aims to improve the lives of children and their families by making decisions about the care of children at risk of significant harm (public law matters), and by regulating disputes between private individuals regarding arrangements for children (private law matters). In public law cases, the State issues care proceedings when concerns about children are so great that parental rights must be curtailed or permanently removed. The court can decide that other family members, foster carers, or adopters should care for children if parental capacity does not improve. In private law cases, parents or other carers who cannot agree on arrangements apply to the court for an order, typically following divorce or separation and concerning with whom a child should live and the time a child should spend with a non-resident parent [1].

Despite this pivotal role, far too little is known about whether the family justice system is delivering the best possible outcomes for children and families, and/or whether policy and legislative change is effective [2]. The lack of knowledge about the family justice system is a long-standing problem and reflects the very limited access to and analysis of core population-wide family justice datasets, either by academics or by government analysts. Whereas progress has been made in using children’s services administrative data in a number of international contexts [3], studies which include family court data are extremely scarce [4]. However, there is growing awareness that more needs to be done to ensure that highly consequential decisions in the family courts are informed by empirical evidence, alongside other forms of knowledge [5].

The Nuffield Family Justice Observatory (Nuffield FJO) [6,7] has, therefore, been established by the Nuffield Foundation to increase understanding of the experiences of children and families in, at the margins of, and beyond the family justice system. Its overarching aim is to bring about change in policy and practice, by providing and improving access to and use of data and research evidence in the family justice system in England and Wales. The vision for the new Observatory was set out in 2018, emphasising the critical place of robust empirical evidence in policy and practice development [4].

As an integral arm of the Nuffield FJO, a data partnership has been established (hereafter, Family Justice Data Partnership (FJDP)) comprising an interdisciplinary team of researchers from Swansea and Lancaster Universities [8]. The FJDP aims to provide a bespoke data platform and analytic support to accelerate use of core family justice data. The partnership will embed national data sources from both England and Wales into the Secure Anonymised Information Linkage (SAIL) Databank at Swansea University, United Kingdom. SAIL has already brought together a wealth of routinely collected population-scale data on health, education, governmental and social care relating to individuals living in Wales, so that they can be reliably and anonymously linked at the individual level and securely used for research [9]. The addition of family justice data within the SAIL Databank enables increased access to core national family justice administrative data and, for the first time, the potential for rich and novel studies through the record-level linkage of these administrative data.

In this article we provide an overview of the content, structure and characteristics of the family justice data, including the details of data transfer and the record matching success. We report on the numbers of applications and individuals (children and adults) involved in family court proceedings.

Furthermore, we report two sets of initial data linkage results by linking the records for individuals from the family justice data with electronic health records (example use one), and property and area level measures of deprivation (example use two). The aim of these two analyses is to demonstrate the potential research opportunities through the use of data linkage techniques using the SAIL Databank rather than to answer any substantive questions about associations with health and deprivation. We discuss data limitations and the potential for additional research possibilities. Although the data we present derive from Wales, the broader methodological points we make and demonstration of linkage, are of broader national and international relevance. Family justice is comparable in the US, Australia, Canada and New Zealand, with all sharing in an aspiration for evidence informed family justice policy and practice [10].

## Methods

### Context

SAIL data are housed in a data repository surrounded by a suite of physical, technical and procedural control measures, which taken together comprise a robust, proportionate governance model with privacy-by-design [11]. The SAIL Databank is hosted and managed on the UK Secure Research Platform (SeRP UK). SeRP UK is an ISO27001 approved customisable technology and analysis platform with a range of functionalities [12]. All uses of SAIL data must be approved by an independent Information Governance Review Panel (IGRP) before data can be accessed [7].

The first family justice data acquired by the SAIL Databank though the work of the FJDP was from Cafcass Cymru, an organisation within Welsh Government’s Health and Social Services group. Cafcass Cymru specialist social workers, or ‘Family Court Advisors’ advise on best interest decisions for children in family court proceedings. Data are collected as part of routine service delivery operations, and comprises information relating to family court proceedings when Cafcass Cymru are involved.

The main data protection legislation applicable in the UK is the UK Data Protection Act (2018) in line with the EU General Data Protection Regulation (GDPR, 2016). By working with a Trusted Third Party to anonymise personal demographic data, SAIL does not receive identifiable details. SAIL relies on the GDPR provisions for processing data for scientific research in the public benefit in order to anonymise de-identified data for research studies.

### Integration with the SAIL Databank

Data are anonymised in the SAIL Databank [9,13] through a succession of processes beginning with a split file process whereby the commonly recognised identifiers (including name and address) are separated from the content data (such as, test results or diagnoses for health data, or legal applications and orders for family justice data). A trusted third party carries out data matching [9] and allocates a unique anonymised linking field (ALF) [14] to each individual represented in the dataset where a successful match is made. The matching algorithm uses deterministic^1^ matching using UK NHS (National Health Service) numbers or full matching of all other identifiers (names, sex, date of birth and post code) followed by probabilistic^2^ matching using a combination of the identifiers. The ALF allows reliable linkage across SAIL datasets. Cafcass Cymru data does not contain NHS numbers so comparison of match rates against data from health based providers is difficult. We present data linkage matching results by linking method and confidence bands for probabilistic matches.

Records successfully matched have an ALF and can be further linked across multiple data sources within SAIL, such as hospital admissions and address registration datasets which facilitate address and area level analyses using a residential anonymised linking fields (RALF) [15] and Lower Layer Super Output Areas (LSOA) linked to area level deprivation indices. Further controls are enacted within SAIL to provide data access for research in anonymised form within a privacy protecting safe haven [9].

### Cafcass Cymru data

Cafcass Cymru records data on public and private family court proceedings involving children where there are concerns over the current or potential welfare of at least one child. It is important to note that as with other case management records, data only covers an individual’s involvement within the family justice system for the period in which the organisation (Cafcass Cymru) were also involved) [16].

The Children Act 1989 requires the appointment of a Children’s Guardian in every public law application under s.31 of the Act – care and supervision proceedings. The Guardian from Cafcass Cymru must remain involved in the case for the duration of care proceedings (although he/she may not attend all hearings). Beyond care proceedings, Guardian involvement is variable. For example, only in cases of contested adoption would the Guardian typically be involved. Cafcass Cymru however receives a number of other public law applications, such as applications to discharge care orders, and revocation of placement orders. It is also important to note that where parents reach voluntary agreement regarding the accommodation of a child under s.76 of the Social Services and Well-Being (Wales) Act, this agreement is made outside of the family courts and hence the Children’s Guardian, and therefore Cafcass Cymru is not involved.

Private family law applications concern disputes between individuals (as opposed to public law proceedings which involve the local authority) who ask the court to make decisions about any aspect of parental responsibility (PR), such as where a child lives, who they see, where they go to school, what medical treatment they have or what religion they follow. The majority of cases are between two parents, although estimates are that approximately 10-15% are non-parent cases [17]. Cafcass Cymru are involved in work with all private law applications prior to and including a first court hearing, thus generalisability of results is not a concern for this initial stage of the court process. However, it should be noted that Cafcass Cymru are only involved in subsequent hearings in specific instances, such as where concerns exist over child welfare and the court has directed further work or has decided to appoint a Children’s Guardian under 16.4 of the Family Procedure Rules. It is also important to note that in the majority of disputes about contact and residence, families do not seek the involvement of the courts, resolving matters on their own or through mediation [18].

- Local authority and the court involved;
- Type of case: public or private;
- Applications: type, date of receipt of case, role of person in proceedings (Applicant[Fn fn-3]3, Subject^4^, Respondents^5^, Others^6^), completion date, application law type;
- Court hearings;
- Legal outputs (procedural directions, orders etc.): date made, type of legal output, application type and to whom the output relates;
- Work carried out by Cafcass Cymru for the court: reports/addendum, safeguarding letters^7^, position statements;
- If any, the type of experts involved as well as solicitors.

The raw Cafcass Cymru ‘Service User’ table contains identifiable data such as names and addresses for the individuals (adults and children) involved in court proceedings; this table was used for the split-file process, with the identifiable data being replaced with an Anonymised Linkage Field (ALF). Records with a successful match based on the identifiable data had an ALF generated, those without a successful match do not have an ALF and we report this match rate (proportion of records linked) by a number of characteristics. Subsequent analyses using linked Cafcass Cymru data are limited to those individuals with an ALF.

### Data Processing

Structured Query Language (SQL) was used for Cafcass Cymru data transformation and preparation to create a series of tables for the analyses contained in this article, which generally cover the start of an individual’s journey in the Family Courts. Other Cafcass Cymru tables can be used to analyse the latter parts of the process such as the final family court order outcomes. Appendix 1 contains a schematic of the Cafcass Cymru and SAIL data used in this analysis.

A standalone dataset was created to analyse the total number of applications by law type and to calculate the match rate for all the individuals (children and adults) involved with the applications over time. The match rate was calculated by dividing the number of individuals who were successfully linked and had an ALF assigned by the total number of individuals. All public law applications received between 2011 and 2019 and private law applications between 2005 and 2019 were included in the dataset. The discrepancy in date coverage is due to legacy data recording methods, meaning that earlier data for public law are not available.

### Cohort Creation

For the purposes of the analyses reported in this article, we created three cohorts. The first cohort was created to allow analysis of the Cafcass Cymru data, to investigate the content of the dataset by law type, sex and age; all children and adults are retained in this cohort. The second and third cohorts were created for demonstration of the potential future analyses based on data linkage to other datasets held in the SAIL Databank, using the individual and residential data linkage keys.

Our cohorts were used to: i) describe the overall individual level Cafcass Cymru data; ii) describe available linked health data for the individuals with a successful ALF linkage; and iii) describe those adults and children with a successful ALF and RALF linkage at area level deprivation. Datasets for each cohort were designed at a unique individual level with one row per individual; duplicates were removed, retaining the most recent Cafcass Cymru application receipt date for analysis. Age was calculated at the application receipt date. Assignment of an individual (adult or child) to either public or private law was based on the following:

- Cafcass Cymru application receipt date.
- Law type indicator held in the Application table.

The implication of this process of selection (i.e. focusing on the most recent application and type), is that an individual could be classed as public law in our cohort, but have previously been involved in a private law case. Some simplification of the data was required for demonstration purposes. Simplification, however, potentially misses the extent of an individual’s full engagement with the family justice system as a minority of individuals (child or adult) will have appeared in both public and private law cases [1].

#### Cohort 1: Cafcass Cymru individual level data

The Cohort 1 inclusion criteria included individuals with public law applications received between 2011 and 2019, and private law applications between 2005 and 2019. Contact ID should be a unique personal identifier relating to only one individual within the Cafcass Cymru Service User table. However, multiple Contact IDs, associated with the same ALF, were identified for a minority of individuals. These 2,268 records (2%) were merged to a single ID, thus removing potential duplication of individuals, but importantly, retaining all linked Cafcass Cymru data for each individual.

#### Cohort 2: Matched individual level health data linkage

Cohort 2 includes only individuals from Cohort 1 with a matched ALF. Given the aim was to link Cafcass Cymru records to health records within the SAIL Databank, we could not include records that had not generated an ALF. A variable was created to flag if an individual had any health record in the two years before or after the application receipt date. We used these to calculate the following proportions (not rates):

- Hospital admissions: admission for any reason and emergency admissions;
- Emergency Department: attendances for any reason;
- Outpatient: appointments for any reason;
- General Practice: any record in dataset regardless of reason.

#### Cohort 3: Matched individual, residential and area level data linkage

Individuals from Cohort 2 were linked to the Welsh Demographic Service Dataset (WDSD) which contains demographic and address level data for individuals registered to a General Practitioner (GP) in Wales. All records were retained where an individual was present in the WDSD at time of the Cafcass Cymru application received date, indicating that they were resident in Wales at that time. The third cohort (Cohort 3) was then defined for individuals where the matching process returned a RALF and a valid LSOA. Cohort 3 was linked to the Welsh Index of Multiple Deprivation tables (WIMD 2014) [19] to obtain income domain deprivation quintiles, which were examined by law type.

## Results

### Application volumes, selection dates and match rates

**Figure 1a and 1b Cafcass Cymru Application Volumes and SAIL Match Rates:**
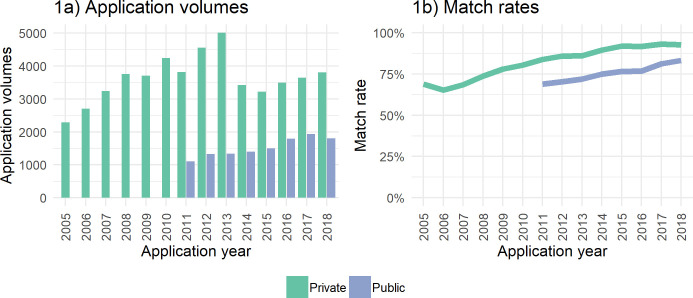


Figures 1a and 1b show the total volume of annual Cafcass Cymru applications alongside SAIL match rates for all individuals (children and adults) involved in all applications for both public and private law^8^. At time of these analyses, only partial data was available for 2019 and therefore excluded. Annual data updates are scheduled going forward.

Following analysis of the applications volume trends by law type, and liaison with Cafcass Cymru regarding data completeness and quality, only records satisfying the following criteria were retained:

- Public Law: application receipt year between 2011 and 2018;
- Private Law: application receipt year between 2005 and 2018.

There were 2,292 private law applications in 2005 increasing to 5,010 in 2013 before decreasing to 3,806 applications in 2018. Public law applications have steadily increased from 1,105 in 2011 to 1,799 in 2018.

The overall match rate was 80%, with rates increasing over time with private law match rates exceeding those in public law by around 10%. We discuss match rate in more detail in later sections and describe variation by a number of factors. The overall match rate compares well with those seen in similar cohorts provided by non-health based organisations. For example, a pilot study for people in a homelessness prevention support programme [20] reports similar match rates and also shows rates improving over time and variation by multiple factors. The results for the proportions and type of data linkage (deterministic or probabilistic) for the Cafcass Cymru data are shown in Appendix 2.

### Cohort analyses

**Figure 2: Cohort Creation Flow Chart:**
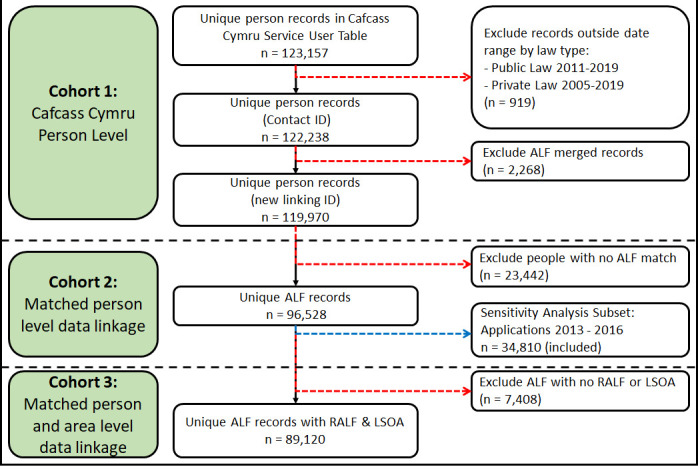


Figure 2 shows total numbers of records at each stage through the data preparation process from raw Cafcass Cymru data to the creation of datasets for each of the three cohorts. The ‘Service User’ table contains 123,157 records, which were reduced to 122,238 following application receipt date and law type exclusion rules. Individual record conflation using the ALF resulted in a final sample size of 119,970 individuals for Cohort 1 with each individual’s most recent application retained for analysis. Cohort 2 consisted of 96,528 individuals with a matched ALF; and the related sensitivity analysis subset contained 34,180 ALFs with application receipt dates between 2013 and 2016. Cohort 3 contained 89,120 individuals, all with a matched ALF, RALF and LSOA.

The majority of exclusions occur between Cohort 1 and Cohort 2 where only individuals with an ALF are retained for further data linkage.

### Cohort 1: Cafcass Cymru individual level data

Table 1 shows the total number of individuals and match rates for a number of Cafcass Cymru data variables. Individuals involved in private law have higher match rates than those in public law (82% and 74% respectively). Public law match rates by ‘person role’ range from 76% to 78% for ‘Subjects’, ‘Respondents’ and ‘Applicants’ with a lower rate of 47% for ‘Others’. There was a wider range for ‘person role’ in private law with 89%, 74% and 83% respectively, ‘Others’ had a lower match rate at 64%.

**Table 1: Cohort 1 characteristics, volumes of individuals and match rates:** 

Grouping		Combined Records		Public Law		Private Law	
			
		Total Number of Individuals	Match Rate (%)	Total Number of Individuals	Match Rate (%)	Total Number of Individuals	Match Rate (%)
Sex	Male	59,026	81	14,635	72	44,391	84
	Female	59,309	82	13,800	78	45,509	83
	Missing	1,635	16	354	19	1,281	15
Age Category	Under 1 year	4,222	68	2,665	60	1,557	80
	1 to 5 years	18,192	85	4,121	75	14,071	88
	6 to 10 years	16,293	89	3,046	88	13,247	90
	11 to 17 years	9,872	89	2,814	88	7,058	89
	18 to 25 years	10,804	86	2,870	88	7,934	85
	26 to 35 years	25,289	85	5,032	85	20,257	85
	36 to 45 years	18,709	85	3,388	83	15,321	85
	46 to 55 years	6,605	85	1,440	82	5,165	86
	over 55 years	2,536	85	593	86	1,943	85
	Missing	7,448	6	2,820	9	4,628	4
Role in Case	Subject	48,188	86	12,400	78	35,788	89
	Respondent	39,493	75	12,102	76	27,391	74
	Applicant	28,667	83	1,782	77	26,885	83
	Other	3,622	53	2,505	47	1,117	64
Total		119,970	80	28,789	74	91,181	82

Match rates are lower for males than for females in public law (72% and 78% respectively). Marked differences exist across age profiles with an average match rate of 86% for those aged over 5 years regardless of law type; children aged 1 to 5 years have higher match rates in private law (88%) compared to public law (75%). Individuals classified as a role type of ‘Others’ on an application also have lower match rates at 47% for public law and 64% for private applications; we discuss potential reasons for this low match rate in the discussion.

**Figure 3: Infant application volumes and match rates for Care and Placement Orders:**
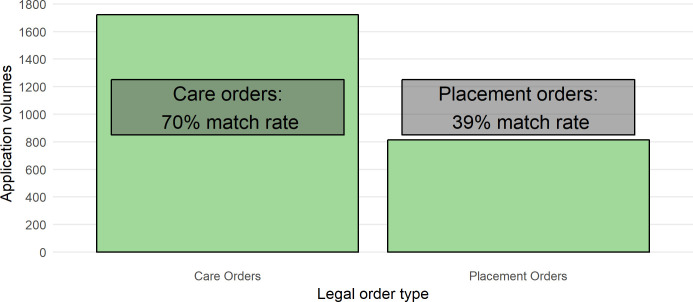


The lowest match rates are seen in infants (those aged less than 1 year) with a 60% match rate in public law, and 80% in private law. Further analysis into the match rates for children involved in public law applications was completed due to the low match rate. We analysed match rate by the application type, using the ‘primary application type’ from the Cafcass Cymru Applications table after categorising applications into three categories: ‘Care orders’^9^, ‘Placement orders’^10^, and ‘Other orders’. The results in Figure 3 show that match rates were 70% and 39% for ‘Care orders’ and ‘Placement orders’ respectively (‘Other Orders’ had a 67% match rate but were not displayed due to low numbers, n=130).

### Cohort 2: Matched individual level health data linkage

Table 2 shows the results of the linking to health datasets held in SAIL Databank for the 96,528 individuals in Cohort 2. The analysis of Cohort 2 was intended as a demonstration of the ability to link the children and adults involved in the family justice system with various health datasets as a proof of concept to enable further research on these populations to be carried out. It also allows the reader to understand the proportions of individuals who have interactions with health services in Wales in the two years prior to, or after the application date. However, it should be clear that the method does not allow comparisons of rates against other populations.

Forty per cent of Cohort 2 had a hospital inpatient admission recorded within the four year observation window; 27% of Cohort 2 had emergency inpatient admissions. Fifty-four percent had an emergency department attendance and 61% an outpatient appointment during the same four year time period. Eighty-four percent of Cohort 2 had at least one record in the GP dataset. The 16% of Cohort 2 without GP data is likely due to individuals attending GP practices that do not provide data to SAIL.

**Table 2: Proportion of Cohort 2 with health event records:** * The SAIL Databank held health datasets have wider date coverage than used in these analyses, for further details see: https://saildatabank.com/saildata/sail-datasets/

Health Data Source	Date and Population Coverage*	Measure(s)	Cohort 2: Proportion of individuals with at least one Health Event
Hospital Admissions (Patient Episode Database for Wales)	- 2003:2018 - All Wales	Admission: 'any reason'	40%
		
		Admission: 'emergencies'	27%
	
Emergency Department (Emergency Department Dataset)	- 2009:2018 - All Wales	Attendance: 'any reason'	54%
	
Outpatients (Outpatient Database for Wales)	- 2004:2018 - All Wales	Any booked appointment	61%
	
General Practice (Welsh Longitudinal General Practice)	- 2003:2018 - 80% Of Practices in Wales	Any record present	84%

### Cohort 3: Matched individual, residential and area level data linkage

**Figure 4: WIMD income deprivation quintile by law type for unique individuals with ALFs with LSOA available (Cohort 3; N = 89,120):**
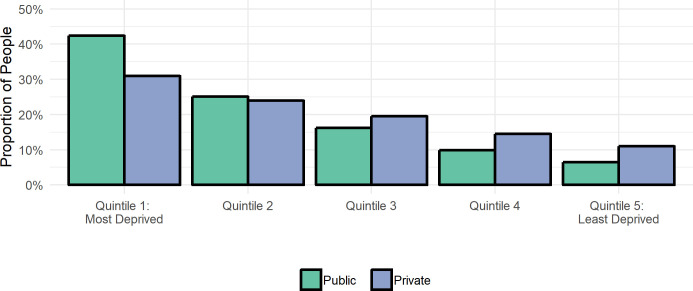


Figure 4 shows where individuals involved in the Family Court proceedings live in terms of area of deprivation and allows comparison between those involved in public and private law proceedings. Taken as a whole, it is evident that individuals involved in Family Law proceedings are more likely to live in areas of higher deprivation. The trends are similar for both law types, albeit with some differences.

For those involved in public law, proportions range from 42% of individuals living in the most deprived area to 6% living in the least deprived areas. The deprivation gradient for those involved in private law is less steep, although individuals are more likely to live in areas of high deprivation, with a range from 31% (most deprived areas) to 11% (in the least deprived areas).

## Discussion

### Summary of main findings

Cafcass Cymru data are available from 2011 to 2019 for public law applications and from 2005 to 2019 for private law applications; data can be accessed by researchers through the SAIL Databank. There were 12,745 public law applications and 52,023 private law applications received by Cafcass Cymru over these periods. The overall match rate was 80%, with variations observed by time, law types, roles, sex and age. The lowest match rates are for infants in public law. Individuals whose role in the case was defined as ‘Others’ also have low match rates, potentially due to the fact that such individuals are more likely to enter the process at a later date when compared to the main parties involved in an application. Individuals, such as wider family members, can apply to become a party to a case, for example, where they propose to provide care for a child. It is important to understand the full set of reasons for lower match rates and the FJDP research team will work with Cafcass Cymru in an attempt to investigate how they may improve match rates over time.

We have demonstrated the potential of data linkage of children and adults involved in Family Courts in Wales to other health and administrative data within the SAIL Databank with two research example uses. Our first example of linking to health datasets showed that 40% of Cohort 2 had hospital inpatient admissions, 27% had emergency admissions, 54% had emergency department attendances and 61% had outpatient appointments, and that 84% of Cohort 2 had a GP record during a four year time period centred around the date that Cafcass Cymru received the application. Our second example showed that individuals involved in private and public law applications were more likely to reside in deprived areas and allowed direct comparison between these groups.

### Strengths and limitations

This work has demonstrated the use of population level administrative data from Cafcass Cymru and its linkage to a wider range of health and other administrative datasets in the SAIL Databank. This is the first time that such work has been completed across the UK, and internationally, there are no comparable studies, which demonstrate the potential linkage of family court records and health data. The availability of the Cafcass Cymru data in the SAIL Databank opens up a range of research possibilities to better understand, provide evidence and ultimately improve lives of those involved in the family justice system in Wales. This foundational work and learning is also transferable to family justice systems beyond Wales and the UK, given that it demonstrates that a) safe anonymous linkage of family court records is possible and b) the potential scope of such linkage. The UK, the US, Australia, Canada and New Zealand all produce similar sensitive courts records, which are as yet insufficiently exploited (safely) for family justice research.

We have reported individual level data linkage match rates by a number of Cafcass Cymru and demographic factors which may assist in guiding future research and data quality improvements, especially in younger populations where we have identified lower match rates. However, within this exploratory work, we have not quantified bias due to loss of individuals through non-matching of records. For example, from Cohort 1 to Cohort 2 there is a 20% reduction (n=23,442), and a further 6% (n=7,408) reduction from Cohort 1 to Cohort 3. Information bias should be considered and addressed, where possible, in further work.

We have demonstrated that match rates are lowest for applications for infants subject to placement orders, which pave the way for children to be placed for adoption. It is important to highlight that the identity of children who were subject to placement orders remained completely anonymous, given existing controls within the SAIL Information Governance model. The low match rates for this group may have implications for future research studies exploring health, social and other outcomes of this population. One possible reason for the low match rates for this group is that a child’s identity is changed upon adoption. Further, those adopted at a younger age would have generated less health data prior to adoption, which may reduce chances of successful matches. Match rates of children involved in adoption should be considered as a special case and caution is urged on interpretation of results. The match rate reported is a static figure describing the proportion of children with an ALF prior to adoption.

Readers should also be aware that Cafcass Cymru is not necessarily involved in adoption proceedings, past the close of care proceedings in public law. At the close of care proceedings a placement order is made; proposed adopters need to return to court once the placement is settled to apply for an adoption order, granting them formal adoptive status within the law. Cafcass Cymru will not generally hold information relating to adoption proceedings and therefore no new details are transferred to SAIL to allow data linkage based on a new identity. Therefore, at this time, it would be difficult to carry out research using linked data for adopted children using Cafcass Cymru data after the point of adoption; however, the FJDP team are investigating methods to enable future research into adopted children.

Health data linkage results indicate there are sufficient volumes of individuals with health utilisation records to support future research into healthcare utilisation and specific health outcomes for children and families. We have also reported on links between deprivation and Family Court involvement for both public and private law, which to our knowledge is the first time in Wales.

Cafcass Cymru data only covers an individual’s involvement within the family justice system for the period during which that agency is involved. Social Care data or family justice data prior to or post Cafcass involvement is not currently available. However, efforts are ongoing through the FJDP to obtain data describing these pathways, which may be available to other researchers in the future.

Cafcass Cymru data are collected for routine service delivery; general limitations regarding the use of administrative data are described elsewhere [21]. Cafcass Cymru implemented a new database in 2015 and content and quality is expected to have improved beyond this point.

Research using Cafcass Cymru data held in SAIL that are not linked to non-Cafcass Cymru datasets can be used to report results on every individual involved. For studies aiming to investigate characteristics or outcomes of children or their families using data linkage to non-Cafcass Cymru data, for example to health datasets within the SAIL Databank, only individuals with an ALF can be included in analyses and therefore the consideration of match rates is important. We have highlighted variation between groups, including for example lower match rates in males, and for infants within public law. Researchers should therefore be aware of potential biases and implications for generalisability of results. The match rate findings can be used to inform future research study design and protocols, although we accept that other characteristics such as ethnicity may affect match rates and should be taken into account in future research. The FJDP research team has established a feedback mechanism to ensure Cafcass Cymru are made aware of data queries and increased use of this resource could aid improved data quality and match rates.

The work presented here is tailored towards the start of the court proceedings process at the point where applications are received at Cafcass Cymru, so should not be considered as covering the whole scope of Cafcass Cymru involvement. This work is purely descriptive in nature and further work will explore associations, for example with deprivation, in more detail.

### Research opportunities and recommendations

Family justice is a domain in need of an enhanced evidence base on which policy and legislative decisions can be made. The FJDP opens up new research opportunities to provide and enable generation of new evidence through inter disciplinary research into family justice, social and health domains. Data from other domains with particular relevance to family justice, such as criminal justice, are not currently available data within the SAIL Databank. However, the FJDP team is working to widen access to such data to enhance future research possibilities.

Cafcass Cymru data are a complex set of relational tables with multiple approaches available for data preparation, analysis and interpretation. We therefore recommend that research should be completed with input from subject matter experts.

As well as Cafcass Cymru data the FJDP has worked with partners to make Cafcass England data available for research via the SAIL Databank. Bedston [22] describes Cafcass England public law data and is a useful resource for researchers using Cafcass data from Wales or England.

The FJDP can support researchers working in this area. SAIL has established an application process to be followed by anyone who would like to access to the data via the Databank (https://www.saildatabank.com/ application-process).

Co-operation across government departments and agencies is central to successful use of administrative data for the public good and work is ongoing via the FJDP and the SAIL Databank, who are aiming to obtain further linked data to more fully understanding pathways of children and families.

## Conclusion

The FJDP plans to facilitate and complete new research to provide evidence for use by the family justice system, ultimately aiming to improve lives of children and their families. We expect a number of broad themes of research, firstly, studies that capture patterns of child and family interaction with the family courts, and characteristics of those involved given that, at present, there is very limited descriptive evidence with this focus, particularly in private law. Secondly, we envisage research capturing the broader health, educational attainment and wellbeing outcomes for children and families. The research will be supported by the availability of a range of justice, social care, health, education and other data resources deposited in the SAIL Databank. This work demonstrates successful data linkage of Cafcass Cymru data in the SAIL Databank and can act to underpin and facilitate inter disciplinary research projects to improve knowledge surrounding children and families in the family courts. 

## Funding and Acknowledgments

The Family Justice Data Partnership is a collaboration between Lancaster University and Swansea University and is funded by the Nuffield Family Justice Observatory. The Nuffield Family Justice Observatory was established by the Nuffield Foundation, an independent charitable trust with a mission to advance social wellbeing. It funds research that informs social policy, primarily in Education, Welfare, and Justice. It also funds student programmes that provide opportunities for young people to develop skills in quantitative and scientific methods. The Nuffield Foundation is the founder and co-funder of the Nuffield Council on Bioethics and the Ada Lovelace Institute. The Foundation has funded this project, but the views expressed are those of the authors and not necessarily the Foundation. Visit www.nuffieldfoundation.org

This study makes use of anonymised data held in the Secure Anonymised Information Linkage (SAIL) Databank, which is part of the national population data research infrastructure for Wales. We would like to acknowledge all the data providers who make anonymised data available for research. For this piece of work we would especially like to thank Cafcass Cymru.

## Contributors

RDJ and LGJ contributed to the conception and design of this work. RDJ prepared and analysed the data; RDJ and LGJ interpreted all aspects of SAIL health and demographic data and the wider FJDP team contributed to the interpretation of Cafcass Cymru data. RDJ drafted the first iterations of the manuscript. All authors critically reviewed the manuscript, provided important intellectual input, approved the final version and agreed to be accountable for their contributions. KB and DF acquired the study funding, the wider FJDP team worked towards obtaining and hosting the data in the SAIL Databank.

## Ethics statement

An application for access to the Cafcass Cymru datasets and other linked datasets in the SAIL databank was reviewed by an independent Information Governance Review Panel (IGRP), which considers each project to ensure proper and appropriate use of SAIL data. Cafcass Cymru data are held in the core restricted category of the SAIL Databank and therefore the project was also approved by the Cafcass Cymru. This study was approved by the IGRP and access was granted through a privacy protecting safe haven and remote access system. Lancaster University also provided ethical review for the project. 

## Footnotes

1 Deterministic data linkage compares identifier(s) across databases making a link if identifier(s) agree.

2 Probabilistic data linkage compares multiple identifiers across databases and calculates a probability of a successful match.

3 The person who makes an application to court; a local authority in public law and (usually) a parent in private law.

4 Subject refers to the child about whom the application has been made.

5 Those individuals entitled to be aware of the application, for example, those with parental responsibility.

6 Individuals who have registered an interest in the application, who are not the applicant, subject or respondent and are allowed to do so under the relevant legal framework, for example, members of extended family.

7 A short report on any safeguarding concerns, potentially drawing upon checks of police and social services records and brief telephone calls with each adult. Used in private law cases only.

8 Public law application volumes were validated against Cafcass Cymru annual reports; figures for private law are not comparable, as Cafcass Cymru publish annual report volumes rather than application volumes.

9 Care orders: the application requests that the local authority seeks to obtain parental responsibility and the child is likely to be placed into foster care

10 Placement orders: the application requests that the child should be placed for adoption

## Appendices

### Appendix 1: Illustrative schematic of datasets used within this analysis

Appendix 1 shows the tables used in this analysis; it is intended to represent the main elements of the available data in terms of tables, relationships, general content and general structure. It is not an exact replica of the data and should not be used to attempt to reproduce results. Variable names may be omitted or changed. Health datasets are simplified, for example, hospital inpatient admission (PEDW) data are held in multiple datasets, not just the single table as depicted here.

### Appendix 2: Proportions of matched records by record linkage classification

Appendix 2 shows ALF matching type and quality for Cohort 2 (all individual level matched records); the majority (94%) of successful ALF matches were for a deterministic record linkage (DRL) match or probabilistic record linkage (PRL) matching confidence over 90%. Actual numbers for each matching classification were: DRL, n=60,875; PRL with 90% or greater confidence, n=2,990; and PRL with 50% to less than 90% confidence, n=5,693. Cafcass Cymru do not collect NHS numbers so no records are classified as 1: NHS number match according to the MACRAL [14] algorithm.

## Abbreviations

ALF	Anonymised Linking Field
FJDP	Family Justice Data Partnership
LSOA	Lower Layer Super Output Area
Nuffield FJO	Nuffield Family Justice Observatory
RALF	Residential Anonymised Linking Field
SAIL	Secure Anonymised Information Linkage
WIMD	Welsh Index of Multiple Deprivation
